# Supplementary material for: The interactome of intact mitochondria by cross-linking mass spectrometry provides evidence for coexisting respiratory supercomplexes
Source: Mol Cell Proteomics. 2017 Dec 8;17(2):216–32. doi: 10.1074/mcp.RA117.000470 (PMC5795388; doi:10.1074/mcp.RA117.000470)
Supplement: Supplemental Data [file supp_RA117.000470_133922_0_supp_23978_fzffwf.zip › spectra_annotation/mito_spectra_annotation/207-1-30-1-1-1.pdf]

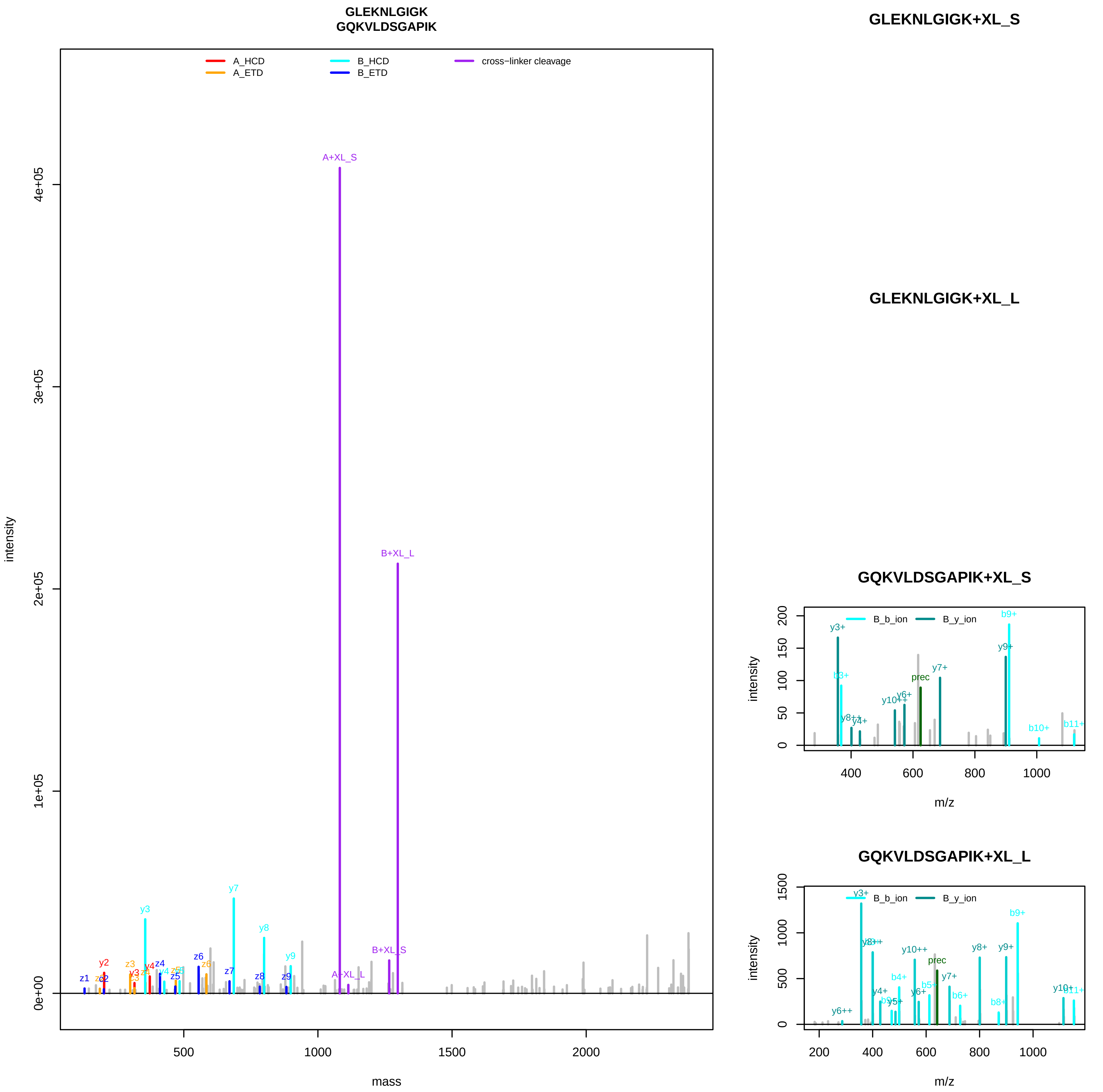
GLEKNLGIGK
GQKVLDSGAPIK
A_HCD
A_ETD
B_HCD
B_ETD
cross−linker cleavage
A+XL_S
B+XL_L
A+XL_L
B+XL_S
y3
y7
y8
y9
z6
z7
z8
z9
z1
y2
z3
y4
z4
z5
intensity
mass
0e+00
1e+05
2e+05
3e+05
4e+05
500
1000
1500
2000
GLEKNLGIGK+XL_S
GLEKNLGIGK+XL_L
GQKVLDSGAPIK+XL_S
B_b_ion
B_y_ion
y3+
b3+
y8++
y4+
y10++
y6+
prec
y7+
y9+
b9+
b10+
b11+
m/z
GQKVLDSGAPIK+XL_L
y6++
b4+
b5+
y5+
y8+
b6+
b8+
y10+
